# Medicinal herb use among asthmatic patients attending a specialty care facility in Trinidad

**DOI:** 10.1186/1472-6882-5-3

**Published:** 2005-02-15

**Authors:** Yuri N Clement, Arlene F Williams, Derick Aranda, Ronald Chase, Nadya Watson, Rochelle Mohammed, Odia Stubbs, Deneil Williamson

**Affiliations:** 1 Faculty of Medical Sciences, The University of the West Indies, St. Augustine, Trinidad and Tobago

## Abstract

**Background:**

There is an increasing prevalence of asthma in the Caribbean and patients remain non-compliant to therapy despite the development of guidelines for management and prevention. Some patients may self-medicate with medicinal herbs for symptomatic relief, as there is a long tradition of use for a variety of ailments. The study assessed the prevalence of use and the factors affecting the decision to use herbs in asthmatic patients attending a public specialty care clinic in Trinidad.

**Methods:**

A descriptive, cross-sectional study was conducted at the Chest Clinic in Trinidad using a *de novo*, pilot-tested, researcher-administered questionnaire between June and July 2003.

**Results:**

Fifty-eight out of 191 patients (30.4%) reported using herbal remedies for symptomatic relief. Gender, age, ethnicity, and asthma severity did not influence the decision to use herbs; however, 62.5% of patients with tertiary level schooling used herbs, p = 0.025. Thirty-four of these 58 patients (58.6%) obtained herbs from their backyards or the supermarket; only 14 patients (24.1%) obtained herbs from an herbalist, herbal shop or pharmacy. Relatives and friends were the sole source of information for most patients (70.7%), and only 10.3% consulted an herbalist. Ginger, garlic, aloes, shandileer, wild onion, pepper and black sage were the most commonly used herbs.

**Conclusions:**

Among patients attending the Chest Clinic in Trinidad the use of herbal remedies in asthma is relatively common on the advice of relatives and friends. It is therefore becoming imperative for healthcare providers to become more knowledgeable on this modality and to keep abreast with the latest developments.

## Background

Recent reports from the Caribbean suggest that the incidence of asthma is following the global trend of increasing prevalence. In Jamaica, a prevalence of 20.8% for exercise-induced asthma was estimated in a cross-sectional study in schoolchildren [1]. About one in ten patients attending an Accident and Emergency Department in Trinidad were treated for acute severe asthma [2] and over 15,000 patients attended four A&E departments throughout the island over a 12-month period [3].

Inhaled corticosteroids as prophylaxis and 'as required' bronchodilator for symptomatic relief are established modalities for asthma management and prevention and the Commonwealth Caribbean Medical Research Council/Global Initiative for Asthma guidelines were adopted in the Caribbean in 1997 [4]. It has been noted that inefficient management predisposes patients to frequent hospitalization and reduced quality of life. In Trinidad, non-compliance and inadequate inhaler technique negatively impact on effective disease management [5,6]. The frequent unavailability of medication at public health facilities and the prohibitive cost at private pharmacies are significantly associated with non-compliance and consequently poor disease control. In these studies, some patients indicated their use of herbal remedies as an alternative to conventional medicines.

Over the last few decades, a global resurgence in the use of herbal remedies has fuelled the growing multi-billion dollar international trade of botanical products. Many patients, dissatisfied with conventional medicines because they expect permanent cures, believe that herbal remedies are 'natural' and sometimes self-medicate without informing their attending physician.

Although there is a long history of traditional use of medicinal herbs throughout the Caribbean [7,8] few studies were done to assess the prevalence of use. Surveys in Jamaica reported an almost 100% use of herbal teas and remedies by respondents throughout the island [9] and 71% in paediatrics inpatients at the University Hospital [10]. These studies, however, assessed only the lifetime use of medicinal herbs and did not identify their use for any particular disease. In Trinidad and Tobago, the use of 'bush medicine' in diabetic patients attending primary healthcare facilities throughout the island was assessed and although 42% reportedly used herbs, only 24% used this healthcare modality for self-management of diabetes [11]. Another survey conducted at an outpatient surgical facility in Trinidad indicated a lifetime prevalence of 86% among patients [12] for any healthcare issue.

This study was undertaken to assess the extent of use of herbal remedies by asthmatic patients attending a specialty chest clinic in Trinidad for symptomatic relief and to determine the factors influencing the patient's decision to use herbs.

## Methods

The study was approved by the Ethics Committee of the Faculty of Medical Sciences, University of the West Indies, St. Augustine campus and permission to interview patients was granted by the Director of the Chest Clinic of the Ministry of Health, Trinidad and Tobago. The study was conducted over the two-month period June to July 2003.

### Sample and setting

The Chest Clinic was chosen as the source of subjects as this is the only national tertiary level health facility specializing in the management of respiratory diseases. Patients entering the study were physician-diagnosed asthmatics based on self-reporting symptoms of wheezing, chest tightness and nocturnal coughing in the previous year. Patients were recruited by consecutive sampling and the nature and purpose of the study were explained on an individual basis. Those confirming their willingness to participate signed their informed consent and were interviewed using a *de novo*, pilot-tested, researcher-administered questionnaire.

### Interview instrument

The questionnaire assessed demographic data such as age, gender, ethnicity, residential district, education, employment and socioeconomic status. Subjects reported their disease severity as intermittent, moderate or severe as determined by the Global Initiative for Asthma (GINA) guidelines with respect to symptom frequency [4]. Patients also reported their use of herbal remedies, identified the herbs used, the frequency of use, source of herbal medicines and the reasons for the use of herbs.

### Statistical analysis

The sample size was calculated as 185 patients assuming a prevalence of 86% [13] with a confidence level of 95%. Since all variables were categorical, χ^2 ^tests were performed to determine whether there were statistically significant associations between the use of herbs and these variables. The p value was set at <0.05 for statistical significance. The data was analyzed using SPSS for Windows (Version 9.0, Chicago, IL).

## Results

### Demography

During the study period one hundred and ninety one patients consented to participate. The demographic details of the sample are given in Table 1. Patients between 35 and 64 years of age formed the largest portion of the sample (62.3%). There was a significant gender difference with females outnumbering males by a 2:1 ratio, p < 0.01. Most patients were of Asian Indian origin (58.1%) and resided in suburban areas (60.2%). There was a high level of unemployment (30.4%); this could be correlated to primary schooling (seven or less years of formal education) being the highest educational level attained in 52.9% and no formal schooling in 5.2% of the sample population. Income was low, with 42.9% of the sample population earning below US$4,000 per year.

**Table 1:** Demographic details of patient sample

**Factors (n = 191)**	**No. (% of sample)**	**No. (%) using herbs**
**Gender**		
Male	61 (31.9)	19/61(31.2)
Female	130 (68.1)*	39/130 (30.0)

**Age groups**		
16–34	34 (17.8)	11/34 (32.4)
35–50	52 (27.2)	21/52 (40.4)
51–64	67 (35.1)	18/67 (21.1)
≥ 65	38 (19.9)	8/38 (13.8)

**Ethnicity**		
African	45 (23.6)	13/45 (28.9)
Asian Indian	110 (57.6)*	31/110 (28.2)
Mixed	35 (18.3)	14/35 (40.0)
Other	1 (0.5)	0/1 (0.0)

**Asthma severity**		
Intermittent	90 (47.1)	29/90 (32.2)
Mild Persistent	29 (15.2)	9/29 (31.0)
Moderate Persistent	27 (14.1)	7/27 (25.9)
Severe Persistent	45 (23.6)	13/45 (28.9)

### Antiasthmatic drug use

The GINA guidelines were recently adopted in the Caribbean and asthmatic patients are currently treated according to their symptom severity. In our sample population, particularly in patients with moderate and severe symptoms, corticosteroids (controllers) and β_2_-agonists (relievers) were prescribed at very high rates, Table 2. Almost 90% of all patients with moderate symptoms were prescribed drugs in these classes. Almost all patients with severe symptoms were prescribed β_2_-agonists. This high level of prescription and use of β_2_-agonists suggest a lack of symptomatic control in our sample population. Theophylline and anticholinergics were prescribed in both categories of patients, but to a lesser extent.

**Table 2:** Antiasthmatic drug use and self-reported compliance in patient sample

**Drug Use & Compliance**	**Asthma Severity**	
	
	**Moderate Persistent (n = 27)**	**Severe Persistent (n = 45)**
Corticosteroids	24 (88.9)	39 (86.7)
β_2 _agonists	24 (88.9)	44 (97.8)
Anticholinergics	5 (18.5)	13 (28.9)
Theophylline	9 (33.3)	19 (42.2)
Self-reported Compliance	27 (100)	43 (95.6)

### Factors influencing the use of herbal remedies

Gender, age, ethnicity, residential district, employment status, income and asthma severity had no statistically significant effect on the use of herbal remedies within the sample population, Table 3. However, almost two-thirds (62.5%) of patients with tertiary education used herbal remedies for asthma, p = 0.025.

**Table 3:** Socioeconomic details of patient sample

**Factors (n = 191)**	**No. (% of sample)**	**No. (%) using herbs**
**Residential district**		
Rural	27 (14.1)	6/27 (22.2)
Suburban	119 (62.3)*	36/119 (30.3)
Urban	42 (22.0)	15/42 (35.7)
No response	3 (1.6)	1/3 (33.3)

**Highest educational level attained**		
No formal education	10 (5.2)	2/10 (20.0)
≤ 7 years of formal education	101 (52.9)	26/101 (25.7)
≤ 12 years of formal education	64 (33.5)	20/64 (31.3)
> 12 year of formal education	16 (8.4)	10/16 (62.5)*

**Employment status **		
Unemployed	58 (30.4)	19/58 (32.8)
Technical	12 (6.3)	6/12 (50.0)
Professional	16 (8.4)	5/16 (31.3)
Clerical	13 (6.3)	6/13 (46.2)
Vocational	28 (12.6)	5/28 (17.9)
Student	8 (4.2)	4/8 (50.0)
Housewife	23 (12.0)	7/23 (30.4)
Pensioner	33 (17.3)	6/33 (18.2)

**Annual income (US$)**		
≤ 3,999	82 (42.9)	22/82 (26.8)
4000 – 9,999	73 (38.2)	23/73 (31.5)
10,000 – 11,999	12 (6.3)	4/12 (33.3)
12,000 – 19,999	15 (7.9)	7/15 (46.7)
≥ 20,000	9 (4.7)	2/9 (22.2)

### Characteristics of patients using herbal remedies

Most patients (70.7%) using herbs were advised by a relative or friend and only 10.3% sought the advice of an herbalist, Table 4. A cultural/traditional basis was the reason for herbal remedy usage in twenty-one (36.2%) patients and another twelve (20.7%) patients used herbs because they felt that were either 'natural' or 'healthy'. Twelve (20.7%) patients used herbs because they believed that their physician-prescribed allopathic medicines were not working.

**Table 4:** Characteristics of patients using medicinal herbs (n = 58)

**Patient characteristics**	**No. (%) (Out of 58)**
**Reason for using herbs**	
Traditional/Cultural	21 (36.2)
Natural/Healthy	12 (20.7)
Conventional medicine not working	12 (20.7)
Other	13 (22.4)
**Source of herbs**	
At home/backyard garden	25 (43.1)*
Supermarket	9 (15.5)
Herbalist/Herbal Shop/ Pharmacy	14 (24.1)
Relative/friend	4 (6.9)
Other	6 (10.3)
**Source of information**	
Relative/friend	41 (70.7)*
Herbalist	6 (10.3)
No consultation	9 (15.5)
Other	2 (3.5)
**Time last used herbs**	
Within last week	17 (29.3)
Within last month	9 (15.5)
Within last 3 months	6 (10.3)
Within last 6 months	3 (5.2)
More than 6 months ago	23 (39.7)

Most patients (58.6%) obtained their herbs or medicinal plants from either their backyards or the supermarket. Only fourteen (24.1%) obtained their herbal supplies from an herbalist, herbal shop or pharmacy. Seventeen (29.3%) of these patients reported using herbs within the last week and most these patients (60.3%) used herbs within the last six months.

Many of these patients were using both physician-prescribed antiasthmatic drugs and herbal remedies, Table 5. No patient with either moderate or severe symptoms indicated that herbal remedies alone were sufficient to relieve symptomatic episodes. It is interesting to note that most patients with moderate symptoms (57.1%) believed that concurrent use of conventional medications and herbs gave better symptomatic relieve. One the other hand, most patients with severe symptoms (53.8%) believed that physician-prescribed medications worked better than herbal remedies, while 23.1% believed that neither relieved their symptoms.

**Table 5:** Antiasthmatic drug use and self-reported compliance in patients using herbal remedies

**Drug Use & Compliance**	**Asthma Severity**	
	
	**Moderate Persistent (n = 7)**	**Severe Persistent (n = 13)**
Corticosteroids	6 (85.7)	10 (76.9)
β_2 _agonists	6 (85.7)	13 (100)
Anticholinergics	1 (14.3)	5 (38.5)
Theophylline	2 (28.6)	7 (53.8)
Self-reported Compliance	5 (71.4)	8 (61.5)
**Subjective Benefits of therapy**		
Herbal remedies alone better	0 (0)	0 (0)
Herbal remedies and drugs better	4 (57.1)	3 (23.1)
Drugs alone better	3 (42.9)	7 (53.8)
Neither herbs and/or drugs work	0 (0)	3 (23.1)

### Herbs used in asthma

Most patients in the sample used more than one medicinal herb simultaneously, which were usually prepared and administered as mixtures in teas. Almost one in four patients using medicinal herbs (22.5%) used either garlic (*Allium sativum*) or ginger (*Zingiber officinale*) for symptomatic relief of asthma, Table 6. Aloes (*Aloe vera*) shandileer (*Leonotis nepetifolia*), wild onion (*Hymenocallis tubiflora*), pepper (*Capsicum spp*.) tulsi (*Ocimum gratissimum*), black sage (*Cordia curassavica*), shadon beni (*Eryngium foetidium*), lemongrass (*Cymbopogon citratus*) and nutmeg (*Myristica fragrans*) were the more popular traditional indigenous West Indian medicinal plants used. Two patients reported using marijuana (leaves and roots). Herbs of European and North American origin, identified as Echinacea (*Echinacea purpurea*), Golden Seal (*Hydrastis canadensis*) and Chamomile (*Matricaria chamomilla*) were less frequently used. Five patients reported using trade name imported tablets for asthma.

**Table 6:** Medicinal plants commonly used by respondents (n = 58) using herbal remedies, ranked by prevalence

**Common name**	**Botanical name**	**n**	**%**
Garlic	*Allium sativum *L.	13	22.4
Aloes	*Aloe vera (Aloe barbadensis *Miller)	13	22.4
Ginger	*Zingiber officinale *Roscoe	9	15.5
Shandileer	*Leonotis nepetifolia *(L.) R.Br.	8	13.8
Wild Onion	*Hymenocalis tubiflora*	8	13.8
Pepper	*Capsium spp*. L.	6	10.4
Black Sage	*Cordidia cylindristachya *R.S.	6	10.4
Tulsi	*Ocimum grastissimum *L.	5	8.6
Echinacea	*Echinacea purpurea *L. Moench	5	8.6
Shadon beni	*Eryngium foetidium *L.	5	8.6
Nutmeg	*Myristica fragrans *Houtt.	4	6.9
Lemongrass	*Cymbopogon citratus *(DC.) Stapfl	4	6.9
Christmas bush	*Chromolaena odorata *(L.) R.M. King & H. Rob.	4	6.9
Golden Seal	*Hydrastis canadenesis *L.	3	5.2
Bayleaf	*Pimenta racemosa *(P. Mill) J.W. Moore	3	5.2
Charmomile	*Matricaria chamomilla *L.	3	5.2
Hibiscus	*Hibiscus rosa-sinensis *Linn.	3	5.2
Noni	*Morinda citrifolia *Linn.	3	5.2
Marijauna	*Cannabis sativum *L.	2	3.5

### Effect of income and education on the use of herbs

Patients using easily accessible herbs such as ginger (*Zingiber officinale*) and aloes (*Aloe vera*), and traditional indigenous medicinal herbs such as shandileer (*Leonotis nepetifolia*) and tulsi (*Ocimum gratissimum*) were more likely to be earning less than US$12,000, Table 7. Herbs of European or North American origin (*Echinacea purpurea *and *Matricaria chamomilla*) were more likely to be used by patients earning in excess of US$12,000 per annum. Income did not affect the use of either garlic or cocoa onion.

**Table 7:** Income and education effects on use of herbs

**Medicinal herb used**	**Percentage of patients with annual income**		**Percentage of patients with formal education**	
	
	≤ US$12,000	> US$12,000	≤ 12 years	> 12 years
Ginger (*Zingiber officinale*)	18.4*	0.0	14.0	20.0
Garlic (*Allium sativum*)	22.5	22.2	16.7	50.0*
Aloes (*Aloe vera*)	24.5*	11.1	25.0	10.0
Shandileer (*Leonotis nepetifora*)	16.3*	0.0	14.6	10.0
Cocoa Onion (*Hymenocalis tubiflora*)	10.2	11.1	10.4	10.0
Tulsi (*Ocimum grastissimum*)	10.2*	0.0	10.4	0.0
Golden Seal (*Hydrastis canadenesis*)	6.1	0.0	6.3	0.0
Echinacea (*Echinacea purpurea*)	4.1	33.3*	4.2	30.0*
Chamomile (*Matricaria chamomilla*)	2.0	11.1*	3.9	10.0

Aloes (*Aloe vera*), tulsi (*Ocimum gratissimum*) and golden seal were preferred in patients with at least twelve years of formal education, Table 7. Garlic and Echinacea were the preferred herbal medicines in patients with more than twelve years formal education. Educational level did not affect the patients' decision to use shandileer (*Leonotis nepetifolia*), wild onion (*Hymenocallis tubiflora*) or ginger (*Zingibe officinale*).

## Discussion

This is the first study of its kind in the Caribbean to assess the use of medicinal herbs by asthmatic patients attending a specialty care clinic. The findings of this study are instructive as the use of medicinal herbs for self-medication in disease management has far reaching implications on the quality of healthcare delivery [14]. We report a prevalence of 30.4% in our patient sample, which is significantly higher than that in the UK, Denmark, Singapore and in the US [15-18].

Most patients using medicinal herbs relied on the advice of relatives and friends as their sole source of information, as were caregivers of children in a US study [19]. We suggest that this information on the use of medicinal plants could have come from traditional/cultural knowledge, anecdotal evidence or from the greater public awareness through information networks such as the internet on the potential medicinal benefits of herbs. Asthma is an emerging chronic disease in the Caribbean and we suggest that the traditional knowledge in this area may be relatively 'new' and exist in relation to other diseases affecting the respiratory tract, such as cough, the common cold and the flu. This may be one of the reasons for the low prevalence of use of herbs in elderly asthmatic patients, as a strong traditional knowledge may not have existed.

We expected a higher prevalence of herbal use in individuals living in rural areas as these districts are depots for traditional knowledge as was reported in Jamaica where rural respondents used a larger variety of herbs than those living in urban areas [10]. As suggested earlier, we suspect that due to the recent emergence of asthma as a chronic disease in the Caribbean it is reasonable to expect that traditional knowledge in the management of this disease is not strong and our results are indicative of this.

We suspected that employment status could have predicted the use of herbs, however, this was not the case in our study sample. Unemployed patients did not improvise more in their use of herbal remedies than those in other income groups, even though most of the herbs used were relatively common, readily available and cheap. The low socioeconomic status of the majority of the sample may have prohibited both consultation with qualified herbalists and the purchase of imported, processed herbs that would have incurred additional out-of-pocket expense to the patient. What we noted was that there was no difference in the use of herbs across the income ranges and that in fact, patients earning relatively modest annual incomes between $US12,000 and $US19,999 were most likely to use herbs, although this did not reach statistical significance.

Attaining a higher education positively influence the decision to use herbs. We suggest that in the absence of traditional knowledge regarding the medicinal use of herbs for asthma, a higher educational level may predispose an individual to greater access to general knowledge, especially with greater exposure to the internet and other sources of information, and this could be a factor in positively influencing the individual's decision to use medicinal herbs. The availability of scientific evidence-based information on the efficacy of herbs for diverse healthcare problems may be particularly significant in patients with the resources to avail themselves to such information, particularly those with higher educational and income levels. This is particularly true for garlic and Echinacea, which have been extensively researched and furthermore patients with higher educational and income levels would be more likely be at an advantage to access information via literature or on the world wide web regarding the use of these medicinal plants.

Patients using imported, processed, and obviously more expensive herbal medications were on the higher end of the socioeconomic scale and were more likely to afford these medications. It was also observed that garlic and Echinacea were the herbs of choice in patients with higher educational levels. These herbs have a long tradition of use and are widely researched in Europe and North America. The traditional use and strong scientific evidence to support their therapeutic efficacy could be important factors influencing the patient's decision. It has been suggested elsewhere that patients with higher educational levels also tend be more involved in the management of their health; they tend to self-medicate or even suggest to their physicians the course of therapy.

Although one in five patients using medicinal herbs stated that "conventional medicines were not working" as the reason for using this alternative healthcare modality, we noted that asthma severity does not affect the decision to use herbs. In previous studies, poor management was associated with non-compliance with prescribed pharmacotherapy and poor inhaler technique [5,6].

The backyard and home garden were major sources of readily available herbs such as aloes, shadon beni and lemongrass. Wild growing 'weeds' such as shandileer, tulsi, cocoa onion and black sage were also identified. The supermarket was a major source of inexpensive common medicinal herbs such as garlic, ginger and nutmeg. The identification of these medicinal herbs provides an opportunity to investigate West Indian plants used to treat asthma to determine whether they possess pharmacological properties. Scientific investigations have shown that some of these herbs possess pharmacological and anti-inflammatory properties, and these may be useful in suppressing the characteristic exaggerated immune response in asthma [20-24]. Pepper and bayleaf have also been shown to exhibit anti-inflammatory properties [[25,26]27]. There is an imperative to commence scientific investigations on traditional West Indian medicinal plants to determine their therapeutic efficacy and safety.

The survey instrument specifically asked questions on the use of medicinal herbs in asthma and did not inquire about the use of herbs as customary teas or tonics. We therefore did not determine lifetime prevalence for the use of herbs in our patient sample, but we suppose that had this been included that there might have been a prevalence similar to those reported in the Jamaica [10,11] and Trinidad [13] surveys. The survey was also limited in that by electing to conduct the study at a public health facility we obviously had a bias towards patients at the lower rung of the socioeconomic ladder, with lower income and educational status. As a consequence, the results reflected patients from this demographic background. We may have expected a different outcome in asthmatic patients attending private institutions, where their characteristics would have been slightly different, as we noted that even in our sample the small number of persons with higher income and educational status tended to use more medicinal herbs for symptomatic relief.

We did not assess whether patients informed their attending physician at the clinic about their use of herbs or determined whether the knowledge or attitudes of these physicians regarding the use of herbs influenced the patients' decision to use herbs. The study was also limited in that we did not ascertain the out-of-pocket expense for herbal remedies by patients, although most stated that herbal medicines (which we supposed were processed, imported products) were more expensive than conventional medicines. We assumed that an additional expense would have only been incurred by those patients purchasing processed, imported herbs obtained from a herbalist, herbal shop or pharmacy (24.1%) and who actually consulted a herbalist (10.3%). We also reasoned that since all the other herbs used were inexpensive and available from either the backyard garden or supermarket (58.6%) that the cost to patients selecting these remedies was minimal.

## Conclusions

The findings of this study are important in that local medicinal plants in Trinidad have been identified in the self-management of asthma in a significant number of patients attending the specialty clinic. These identified herbs can now be targeted for scientific investigation to determine whether their pharmacological efficacy will assist in the development of viable healthcare alternatives in a developing country. These findings are also important for policymakers in the health sector who are given the mandate to regulate issues pertaining to the public's health. We are also becoming more aware of the potential for critical interplay between herbs and drugs when taken concomitantly to produce life-threatening interactions. Since herbs are here to stay and patients will continue to self-medicate with increasing frequency, it is imperative that healthcare providers become more knowledgeable on this modality and keep abreast with the latest developments in herbal therapy.

## Competing interests

The author(s) declare that they have no competing interests.

## Authors' contributions

YNC was the P.I. in this study. He was responsible for the study concept, development of methodology, coordinating the research activities, analyzing the data, and writing the manuscript. AFW was responsible for data input and analysis. DA was involved in methodological development, data collection, data input and analysis and presentation at regional conference. RC was involved in methodological development, data collection, data input and analysis. NW was involved in methodological development, data collection and input. RM was involved in methodological development, data collection and input. OS was involved in methodological development, data collection and input. DW was involved in methodological development, data collection and input. All authors read and approved the final manuscript.

## Pre-publication history

The pre-publication history for this paper can be accessed here:


